# Differential Activation of NRF2 Signaling Pathway in Renal-Cell Carcinoma Caki Cell Lines

**DOI:** 10.3390/biomedicines11041010

**Published:** 2023-03-24

**Authors:** Naomi L. Hitefield, Stephen Mackay, Lauren E. Hays, Shimin Chen, Ian O. Oduor, Dean A. Troyer, Julius O. Nyalwidhe

**Affiliations:** 1 Department of Microbiology and Molecular Cell Biology, Eastern Virginia Medical School, Norfolk, VA 23507, USA; 2 Leroy T. Canoles Jr. Cancer Research Center, Eastern Virginia Medical School, Norfolk, VA 23507, USA

**Keywords:** renal-cell carcinoma, clear-cell renal-cell carcinoma, signaling pathway activation

## Abstract

Renal-cell carcinoma (RCC) is a heterogeneous disease consisting of several subtypes based on specific genomic profiles and histological and clinical characteristics. The subtype with the highest prevalence is clear-cell RCC (ccRCC), next is papillary RCC (pRCC), and then chromophobe RCC (chRCC). The ccRCC cell lines are further subdivided into prognostic expression-based subtypes ccA or ccB. This heterogeneity necessitates the development, availability, and utilization of cell line models with the correct disease phenotypic characteristics for RCC research. In this study, we focused on characterizing proteomic differences between the Caki-1 and Caki-2 cell lines that are commonly used in ccRCC research. Both cells are primarily defined as human ccRCC cell lines. Caki-1 cell lines are metastatic, harboring wild-type *VHL*, whereas Caki-2 are considered as the primary ccRCC cell lines expressing wild-type von Hippel–Lindau protein (pVHL). Here, we performed a comprehensive comparative proteomic analysis of Caki-1 and Caki-2 cells using tandem mass-tag reagents together with liquid chromatography mass spectrometry (LC/MS) for the identification and quantitation of proteins in the two cell lines. Differential regulation of a subset of the proteins identified was validated using orthogonal methods including western blot, q-PCR, and immunofluorescence assays. Integrative bioinformatic analysis identifies the activation/inhibition of specific molecular pathways, upstream regulators, and causal networks that are uniquely regulated and associated with the two cell lines and RCC subtypes, and potentially the disease stage. Altogether, we have identified multiple molecular pathways, including NRF2 signaling, which is the most significantly activated pathway in Caki-2 versus Caki-1 cells. Some of the differentially regulated molecules and signaling pathways could serve as potential diagnostic and prognostic biomarkers and therapeutic targets amongst ccRCC subtypes.

## 1. Introduction

Renal-cell carcinoma (RCC) is currently the eighth leading cause of deaths that are attributed to cancer in the US and in 2022, about 79,000 new cases of renal cancer were diagnosed with about 13,920 deaths resulting from the disease [1]. In 2020, the incidence of the disease was 430,000 new cases globally [2]. RCC is heterogeneous and the disease consists of several histological subtypes based on unique genomic profiles and clinical characteristics [3]. The most prevalent subtype of RCC with approximately 80% of the cases is clear-cell RCC (ccRCC) [4]. The condition is commonly defined by biallelic loss of tumor suppressor genes on chromosome 3p, which includes *VHL*, *BAP1*, *SETD2,* and *PBRM1* [5,6]. The second most common subtype is papillary RCC (pRCC), accounting for about 15% of malignant kidney tumors [7]. Oncogenesis of type I pRCC is associated with the activation of the MER oncogene at 7q31 in addition to amplifications of chromosomes 7 and 17 [8,9,10]. The least prevalent form is chromophobe RCC (chRCC) which accounts for approximately 5% of the cases. Typically, chRCC has a less aggressive disease phenotype compared to ccRCC and pRCC [11,12]. In addition, several other rare subtypes of RCC have been described [11,12]. The availability of biological models with unique tumorigenic phenotypes has been used as platforms for the development of novel interventions against RCC and other cancers. Immortalized tumor cell lines are the most commonly used model systems in cancer research, making a significant contribution to our current molecular and genetic understanding of RCC biology [13,14]. The extensive in vivo heterogeneity of RCC calls for the development and availability of cell lines with diverse phenotypic features to be utilized as models for basic and applied renal cancer research [15]. These studies could play a major role in the discovery, optimization, and translation of treatments from the bench to the bedside [16]. For example, the NCI-60 cancer cell lines panel is routinely used for preclinical drug screening to determine their efficacy. This allows for early triaging of ineffective compounds and their exclusion from subsequent testing and assessments that require animal models and humans [17]. With the observed heterogeneity of RCC disease phenotypes, the ability to correctly discriminate and distinguish between malignant cancer types, including ccRCC, pRCC, ChRCC, and other rare subtypes, is crucial for patient management decisions. A subset of patients diagnosed with ccRCC, approximately 20–30%, remain undiagnosed until the cancer has already metastasized [18,19]. Accurate identification provides prognosis, predicts progression, and determines treatment strategies, including therapeutic options and severe interventions like nephrectomy.

We have performed comprehensive comparative qualitative and quantitative proteomic studies on Caki-1 and Caki-2 cells to better understand their RCC subtypes and disease stage phenotypes. The objective is to identify proteins and uncover molecular pathways that can be used to further discriminate the two cell lines and RCC subtypes. Caki-1 cells were established from a skin metastatic RCC lesion of a 49-year-old Caucasian man, and are a commonly used model for metastatic ccRCC, harboring wild-type *VHL*. These cells develop tumor lesions with clear-cell phenotypes in mice [20]. In addition, Caki-1 cells produce high VEGF levels under hypoxic conditions consistent with the ccRCC phenotype [21,22]. In culture, Caki-1 cells are polarized and exhibit morphological, physiological, and biochemical features of functional differentiated kidney tissue and hence their proposed use as experimental models for proximal tubule epithelium [23]. On the other hand, Caki-2 was isolated and developed from a kidney primary tumor from a 69-year-old Caucasian male and classified as a ccRCC cell line expressing wild-type pVHL, but lacking HIF-2α expression [24]. The reduced HIF1α protein expression in these cells has not been fully explained [24]. Characterization of Caki-2 tumors in nude mice reveals the development of cystic papillary tumors that possess microvilli and microfilaments, and a reduced number of some organelles including mitochondria, lysosomes, and a reduction of lipid droplets and multilamellar bodies [25,26,27]. Caki-2 cells exhibit increased levels of *MET* and *LRRK2* expression [28], and abnormalities in chromosome eight [29], which are consistent with features for pRCC. In addition, there are inconsistencies in *VHL* mutation status in this cell line, with conflicting reports of lack of mutations [24], and detection of mutations on the alpha domain of the VHL tumor suppressor protein [30,31], which would support a clear-cell histological phenotype for these cells. These inconsistencies have led to the dual utilization of Caki-2 cells in ccRCC [21,32,33] and pRCC [26,27,28,34] research. Recent genomic studies have further classified ccRCC cell lines into prognostic groups based on validated ccA and ccB expression-based subtypes [15,35,36]. In these analyses, Caki-1 is classified under ccB, whereas Caki-2 is not predicted to be of either class with high confidence.

Our comprehensive qualitative and quantitative proteomic analysis of the two cell lines uncovered significant differential regulation of specific proteins and activation/inhibition of specific molecular pathways, upstream regulators, and causal networks that are uniquely regulated and associated with the two cell lines and RCC subtypes and, potentially, disease stage. Some of these molecules and pathways, including NRF2 signaling, which is the most significantly activated pathway in Caki-2 versus Caki-1 cells, could serve as potential diagnostic and prognostic biomarkers and therapeutic targets for RCC subtypes.

## 2. Materials and Methods

### 2.1. Materials

The reagents and chemicals for culturing cells, protein isolation, and processing for mass spectrometry analysis were purchased from Thermo Fisher Scientific (Waltham, MA, USA), unless otherwise specified.

### 2.2. Renal Cancer Cell Lines

Two renal cancer cell lines Caki-1 (HTB-46) and Caki-2 (HTB-47), obtained from the American Type Culture Collection (ATCC) (Manassas, VA, USA), were used for the study. Testing confirmed that the cells were not contaminated with mycoplasma. All the experiments were performed using cells with passage number ≤5. The cell characteristics, as provided by ATCC, are given in Supplementary Table S1**.** Three independent biological experiments were performed using the two cell lines. The cells were cultured in McCoy’s 5A Media containing 10% fetal bovine serum, 1× penicillin/streptomycin, 1 mM sodium pyruvate, and 25 mM HEPES buffer at 37 °C in 5% CO_2_.

### 2.3. Protein Isolation and Quantification

For each cell line (Caki-1 and Caki-2), three 100 mm culture dishes were harvested separately via scraping after in plate lysis with a RIPA buffer containing 1× Halt Protease and Phosphatase Inhibitor Cocktail. Concentrations of the triplicate total protein lysates from the two cell lines were determined using a Bicinchoninic Acid (BCA) assay. The lysates were aliquoted and stored at −80 °C before subsequent analysis. For the two cell lines, 100 µg aliquots from the three independent experiments were acetone precipitated overnight at −20 °C. Precipitated protein was collected via centrifugation at 13,000× *g* for 10 min at 4 °C. Pellets were air-dried prior to processing for trypsin digestion and tandem mass tag (TMT) prior to mass spectrometry analysis.

### 2.4. Protein Digestion and TMT Labeling of Peptides

Protein digestion and labeling of Caki-1 and Caki-2 cell lysates were performed using TMT-6plex following the manufacturer’s instructions. In brief, triplicate 100 µg acetone precipitated protein was dissolved in 100 μL 100 mM triethylammonium bicarbonate (TEAB). Proteins were reduced with 10 mM Tris-(2-carboxyethyl)-phosphine (TCEP) at 55 °C for 1 h. Reduced proteins were alkylated with 15 mM iodoacetamide for 30 min at room temperature in the absence of light, and precipitated with acetone for 4 h at −20 °C. Precipitated protein was pelleted via centrifugation at 8000× *g* for 10 min at 4 °C. The pellets were dried briefly, prior to solubilization in 100 μL of 50 mM TEAB, and digestion with trypsin for 16 h at 37 °C at a 40:1 protein/substrate-to-protease ratio. TMT−6plex reagents (800 μg) solubilized in anhydrous acetonitrile were mixed with 100 μg of tryptic peptide at a final concentration of approximately 30% (v/v) acetonitrile for three Caki-1 (TMT tags 126, 127, and 128) and three Caki-2 (TMT tags 129, 130, and 131) labeling reactions. The labeling reactions proceeded for 1 h at room temperature before quenching with 0.3% (v/v) hydroxylamine. The six TMT-labeled peptide samples were all combined in a single microcentrifuge tube at a stoichiometric ratio of 1:1:1:1:1:1 before centrifugation in a SpeedVac to near dryness.

### 2.5. High pH Reversed-Phase Fractionation of TMT Labeled Peptides

For fractionation, 100 μg of the pooled Caki-1 and Caki-2 TMT-labeled peptides resuspended in 0.1% trifluoroacetic acid (TFA) solution and used for high pH reversed-phase (HPRP) chromatography using Pierce HPRP peptide fractionation kits as per the manufacturer’s instructions (Thermo Fisher Scientific, 84868). Peptides were loaded onto columns after equilibration with 0.1% trifluoro acetic acid (TFA). Unbound peptides were collected by adding 300 µL of wash buffer consisting of 5% acetonitrile in 0.1% triethylamine and centrifuging at 3000× *g* for 2 min. Bound peptides were eluted sequentially using 300 µL of eight increasing concentrations of elution buffers with 10.0%, 12.5%, 15.0%, 17.5%, 20.0%, 22.5%, 25.0%, and 50% acetonitrile in 0.1% triethylamine respectively. The wash and eight eluted fractions in individual tubes were evaporated to dryness in a SpeedVac prior to reconstitution for LC/MS analysis.

### 2.6. Liquid Chromatography and Tandem Mass Spectrometry

The peptides were resuspended at a normalized concentration of 0.5 µg/µL in 0.1% formic acid (FA) before analysis on an Orbitrap Fusion Lumos Tribrid Mass Spectrometer (Thermo Fisher Scientific, Waltham, MA, USA)) coupled to an EASY-nLC 1200 UHPLC system. Normalized amounts of peptides were loaded on a C18 pre-column (3 µm particle size, 2 cm length, 75 µm inner diameter, and 100 Å pore size) (Thermo Fisher Scientific, Waltham, MA, USA)), in line with a C18 analytic column (2 µm particle size, 50 cm length, 75 µm inner diameter, 100 Å pore size) (Thermo Fisher Scientific, Waltham, MA, USA)). For these analyses, two micrograms of the wash and eight high pH reversed-phase fractionations were injected and separated by liquid chromatography in line with the mass spectrometer using aqueous 0.1% FA as a buffer (buffer A) and 0.1% FA in 80% acetonitrile as buffer B. The LC running parameters were as follows: flow rate 250 nl/min; isocratic 8% solvent B for 8 min, 8–12% solvent B linear gradient over 5 min; 12–30% solvent B linear gradient over 100 min; 30–60% solvent B linear gradient over 20 min; 60–98% solvent B linear gradient over 5 min; isocratic 98% solvent B over 10 min for a total of 140 min LC/MS/MS run. To minimize carryover, two column washes with buffer A were included after every sample injection in the sequence run. Supplementary Table S2 provides a summary of the parameters for the mass spectrometry instrument method that was used in the analyses.

### 2.7. Data and Bioinformatics Analysis

Mass spectra raw files were processed in Xcalibur and analyzed using Proteome Discoverer version 2.4 (Thermo Fisher Scientific, Waltham, MA, USA)) with the Sequest-HT search engine. Peptide database searching was performed against a fasta file of annotated canonical human proteins comprising 20,291 sequences downloaded from UniProt on 26 January 2022. The searches were also performed against a decoy database comprising all of the protein amino acid sequences in reversed order. Peptide spectral matches identified with a less than 1% false discovery rate (FDR ≤ 0.01) were included for further analysis. The parameters used in the searches were: full trypsin specificity selected with a maximum of two missed cleavage sites; fixed modifications, carbamidomethylation (C), and TMT 6-plex (K and peptide N terminals). Dynamic modifications, oxidation (M), and deamidation (N, Q). Precursor ion mass tolerances, 10 ppm MS acquired in the Orbitrap and MS/MS mass tolerance of 0.6 Da for spectra acquired in the ion trap. The mass spectrometry proteomics data have been deposited to the ProteomeXchange Consortium via the PRIDE [37] partner repository with the data set identifier PXD040083. 

Further MS data analysis for the proteins was performed using Proteome Discoverer to determine peptide spectral matches, protein abundance ratios (Caki-2/Caki-1), t-test *p*-values, and Benjamini–Hochberg adjusted *p*-values, Sequest-HT and PEP scores, % sequence coverage, the sum of all and unique peptides identified. The proteins that were used for comparative analysis had a minimum of two unique high-scoring peptides identified. Differentially expressed proteins between Caki-1 and Caki-2 cells were used for enrichment analysis using different modules that are embedded in Proteome Discoverer 2.5. The proteins that were used in these analyses were filtered to fulfill the following inclusion criteria: o peptides with high Sequest-HT scores; FDRs <1%; proteins with an abundance ratio ≥ 1.5 and *p*-value of ≤0.05. These include gene ontology analysis for molecular function, cellular components, and biological processes. Differentially expressed proteins were submitted for searches using the Kyoto encyclopedia of genes and genomes (KEGG) pathway database [38], the Wiki biological pathway database https://www.wikipathways.org (accessed on 10 January 2023), and the Reactome pathway database (https://reactome.org/(accessed on 10 January 2023). To gain further insights into the molecular differences between the two cell lines, the same proteins were subjected to ingenuity pathway analysis (IPA) (Qiagen Digital Insights, Redwood City, CA, USA) “core analysis” to generate a report consisting of top canonical pathways, upstream analysis (including upstream regulators and causal networks), diseases and functions, regulator effects, and molecular and biological functions of differentially expressed proteins between the two cell lines.

### 2.8. Western Blot Analysis

Forty micrograms (40 μg) of protein lysates from the two cell lines were separated by SDS-PAGE and transferred to PVDF membranes and processed for targeted protein detection, as we have previously described [39]. Briefly, to minimize nonspecific binding, an Odyssey blocking buffer was used to block PVDF membranes for 1 h before incubation with the corresponding primary antibodies and GAPDH as the loading control at 4 °C overnight. Supplemental Table S3 summarizes the vendor, species, and dilution of the primary antibodies that have been used in the study. The membranes were washed for 5 min four times using 0.1% Tween-20 in PBS. Thereafter, membranes were incubated with the corresponding IRDye700 or 800-conjugated secondary antibodies protected from light at room temperature for 1 h. The membranes were subsequently washed extensively using 0.1% Tween-20 in PBS, before detection and imaging of the target and GAPDH protein bands using a LI-COR Odyssey infrared Imager (LI-COR Biosciences, Lincoln, NE, USA).

### 2.9. Immunofluorescence Assay

Caki-1 and Caki-2 cells were cultured in 6-well plates containing glass coverslips and cultured in McCoy’s 5A Media supplemented with 10% FBS for 48 h. Coverslip adherent cells were washed three times using cold PBS before fixation with ice-cold methanol for 5 min. Fixed cells were washed three times with PBS and incubated with CA12 antibodies overnight at 4 °C. The primary antibody solution was discarded before washing the cells three times with ice-cold PBS. The cells were stained with Alexa Fluor conjugated secondary antibodies mixed together with nucleic DNA TOPRO stain at room temperature for 1 h. Secondary antibodies and DNA TOPRO stain were washed away using PBS before mounting the coverslips to glass slides using a mounting medium containing 4′,6-diamidino-2-phenylindole (DAPI) stain (Vector Labs, Burlingame, CA, USA). The coverslips were sealed with nail polish prior to microscopic examination and image capture using a Zeiss 880 confocal microscope (Carl Zeiss, White Plains, NY, USA).

### 2.10. Quantitative PCR Analysis

Total RNA extraction from Caki-1 and Caki-2 cells was performed using the All-Prep DNA/RNA Mini Kit (Qiagen, 80204) (Qiagen, Germantown, MD, USA) as we have previously described [39,40]. The RNA concentration and purity were determined through a NanoDrop One Spectrophotometer and 2100 Bioanalyzer (Agilent Technologies, Wilmington, DE, USA). RNA was converted to single-stranded cDNA and purified using the SuperScript First-Strand Synthesis System for RT-PCR (Invitrogen, 11904–018). qPCR reactions using primers targeting *CA12* (forward 5′-GGACAAATGGGGACAGGAAGGATCAAG-3′ and reverse 5′-GAGGACATTTCATGCTGACAAAATGAG-3′) and *NEFL* (forward 5′-ATGAGTTCCTTCAGCTACGA-3′ and reverse 5′-TCAATCTTTCTTCTTAGCTGCTTGTTC-3′), with *GAPDH* (forward 5′-ACCACAGTCCATGCCATCAC-3′ and reverse 5′-TCCACCACCCTGTTGCTGTA-3′) as the loading control, were analyzed concurrently on a Bio-Rad CFX96 Real-Time System C1000 instrument (Bio-Rad, Hercules, CA, USA). The qPCR analyses were performed in triplicate and included negative control experiments.

### 2.11. Effects of Sulfamate S4 Inhibitor on the Viability of Caki-2 Cells

The S4 inhibitor, a 4-(3′-(3″,5″-dimethylphenyl)ureido)phenyl sulfamate inhibits tumor-associated carbonic anhydrases CA9 and CA12 while avoiding off targeting of the cytosolic CA1 and CA2 enzymes [39,40,41,42,43]. The inhibitory activity is directly dependent on the membrane-associated CA9 and CA1 isoforms [41,44]. The effect of S4 inhibitor treatment on the viability of Caki-2 cells was evaluated by culturing the cells under normoxic or hypoxic conditions in a culture medium containing 33 μM S4 or DMSO for 24 h. After treatment and incubation, cell viability was measured using the Trypan Blue Assay [45].

### 2.12. Statistical Analysis

Comparative statistical analysis was done using GraphPad Prism 9.4.1 (San Diego, CA, USA) and, unless otherwise stated, the results are expressed as mean ± standard deviation. For experimental groups, comparisons were performed using unpaired Student’s t-test and one-way ANOVA with Tukey’s post hoc analysis. The significance threshold for comparisons for all analyses was set at *p* < 0.05. 

## 3. Results

### 3.1. Qualitative and Quantitative Proteomic Analysis of Caki-1 and Caki-2 Cells

A global discovery-based comparative quantitative proteomics analysis strategy using TMT-6plex was utilized to uncover differential protein expression between the two cells. Mass-spectrometry data acquisition was performed on a Tribrid Orbitrap Fusion Lumos instrument using synchronous precursor selection (SPS) MS^3^ fragmentation scans for protein identification and differential quantitative analysis. Proteome Discoverer 2.5 was used to determine abundance ratios (Caki-2/Caki-1), perform statistical calculations to determine *p*-values, adjusted *p*-values, Sequest-HT and posterior error probability (PEP) scores, protein percent sequence coverage, the sum of all and unique peptides identified. In this analysis, a total of 7315 proteins were identified. The identified proteins were filtered based on the criteria described under Section 2.6, which include a minimum of two unique high-scoring peptides, with FDR < 1%, minimum abundance ratio ≥ 1.5, and *p*-value ≤ 0.05. Based on these criteria, 250 differentially expressed proteins (DEPs) were identified between the two cell lines (162 upregulated and 82 downregulated in Caki-2 versus Caki-1 cells). The full list of 250 proteins is provided in Supplementary Table S4. The top 15 differentially expressed genes proteins (up/down) between the two cell lines are shown in Table 1 below.

### 3.2. Western Blot, RT-qPCR, and Immunofluorescence Assay Analysis of Differentially Expressed Proteins

**Figure 1:**
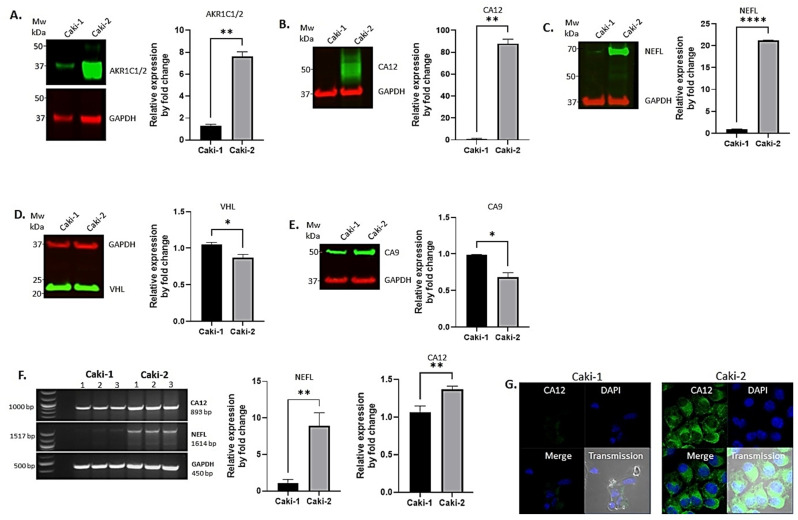
Differential expression of proteins in Caki-1 and Caki-2 cells. (**A**) Western blot detection and validation of expression profiles of target proteins including (**A**) AKR1C1/2, (**B**) CA12, (**C**) NEFL, (**D**) VHL, and (**E**) CA9. GAPDH was used as a western blot loading control. The expression-fold change of the proteins is based on normalization by individual GAPDH values. There is a very significant enhanced expression of AKR1C1/2 (*p* = 0.0023), CA12 (*p* = 0.0011), and NEFL (*p* = 0.0001) in Caki-2 versus Caki-1 cells. The expression levels of VHL and CA9 differ marginally amongst the two cells (*p* ≤ 0.05). (**F**) Semi quantitative mRNA expression of CA12 and NEFL in Caki-1 versus Caki-2 cells. cDNA was synthesized from total mRNA extracted from the two cell lines and used for RT-qPCR to evaluate their relative expression levels in the two cell lines. qPCR reactions amplifying *CA12* (893 b.p.), *NEFL* (1614 b.p.), and *GAPDH* (450 b.p.) in the two cell lines were analyzed via gel electrophoresis on 2% agarose gels containing EtBr for DNA visualization under UV. Relative band intensities were measured and used for quantitation with *GAPDH* as a control for normalization. There is a significantly enhanced expression of *CA12* and *NEFL* transcripts in Caki-2 compared to Caki-1 cells (*p* ≤ 0.05). (**G**) Immunofluorescence confocal microscopy detection of CA12 in the two cell lines. The detection and visualization of CA12 are significantly higher in Caki-2 cells. For the bar graphs, significant differential expression profiles are depicted using asterisks corresponding to the following thresholds * (*p* < 0.05), ** (*p* < 0.005) and **** (*p* < 0.0001).

To validate the mass spectrometry data and provide orthogonal evidence to support our findings, three proteins showing significant differential expression profiles in the two cell lines and relevant to some of our conclusions concerning the metabolic phenotypes of the two cell lines were targeted for -validation. Consistent with the quantitative proteomics data, the immunoblots clearly confirm a significantly increased abundance of AKR1C2, CA12, and NEFL in Caki-2 than in Caki-1 cells using GAPDH as the control (*p* < 0.05) (Figure 1A–C). By contrast, there are minimal expression differences in the expressions of other relevant proteins, including CA9 and VHL, which were not identified as differentially expressed based on the filtering criteria of the MS experiments (Figure 1D,E). The expressions of *CA12* and *NEFL* were also validated by RT-qPCR using *GAPDH* as the control (Figure 1F). Therefore, the observed concordance between the mass spectrometry/proteomics results, western blot, and qPCR data is highly significant and acceptable and supports the utility of the TMT-based quantitative proteomic approach that is utilized in the study. Among the differentially expressed proteins as demonstrated by quantitative mass spectrometry, western blot, and qPCR, we further validated the expression of CA12, which is significantly upregulated in Caki-2 vs. Caki-1. Unlike CA9 expression, which is well characterized in RCC, the expression levels of CA12 have not been comprehensively studied in RCC. Immunofluorescence assays verify the overexpression of CA12 in Caki-2 cells and further confirm the quantitative proteomics results with significant expression of CA12 in Caki-2 versus Caki-1 (Figure 1G).

### 3.3. Sensitivity of Caki-2 Cells to Sulfamate S4 Inhibitor

**Figure 2:**
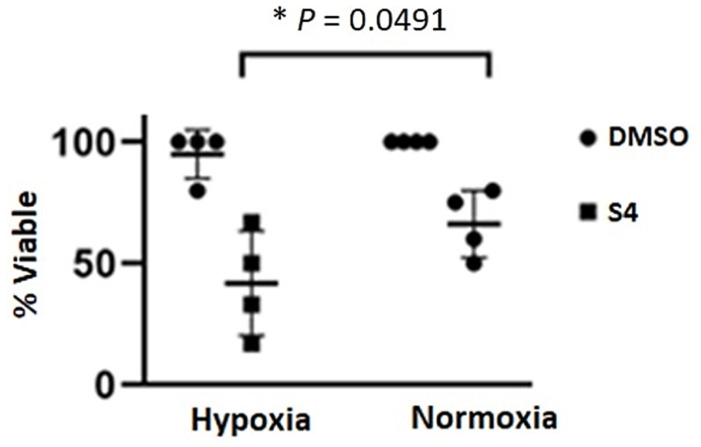
Effect of S4 inhibitor on the viability of Caki-2 cells. Caki-2 cell cultures were treated with 4-(3′-(3”, 5”-dimethylphenyl)ureido)phenyl sulfamate (S4) in normoxic and hypoxic conditions in four replicate experiments. Additional cultures were treated with DMSO as controls. Following incubation, cell viability was assessed using the Trypan Blue assay. DMSO has no effect on the viability of Caki-2 cells, whereas S4 significantly reduces cell viability under hypoxic and normoxic conditions. Cell viability is significantly reduced under hypoxic conditions as demonstrated by one-way ANOVA using Šidák’s multiple comparisons test (*p* = 0.0491). Significantly different cell viability depicted by an asterisk *p* < 0.05.

After validating the significant upregulation of CA12 in Caki-2 versus Caki-1 cells, we utilized in vitro assays to determine the effect of a CA9/CA12 inhibitor, 4-(3′-(3″,5″-dimethylphenyl) ureido) phenyl sulfate (S4) on Caki-2 cells under hypoxic and normoxic conditions. In both hypoxic and normoxic conditions, Caki-2 cells treated with DMSO maintained high levels of viability at 95% and 100%, respectively. The addition of the S4 inhibitor decreased Caki-2 cell viability under normoxic conditions to an average of 66.25% (33.75% dead). Under hypoxic conditions, the S4 inhibitor reduced the viability of Caki-2 cells to an average of 41.75% (58.25% dead). These results indicate that the inhibitor reduces Caki-2 cell viability in both normoxic and anoxic conditions, with a significant decrease under hypoxic conditions (Figure 2). It is important to note that HIF1α protein is detected at very low abundance in Caki-2 versus Caki-1 cells [24] (Supplementary Figure S1). Our in vitro data provides the rationale for future studies targeting CA12 in different RCC subtypes. 

### 3.4. Bioinformatic Analysis of Differentially Expressed Proteins

**Figure 3:**
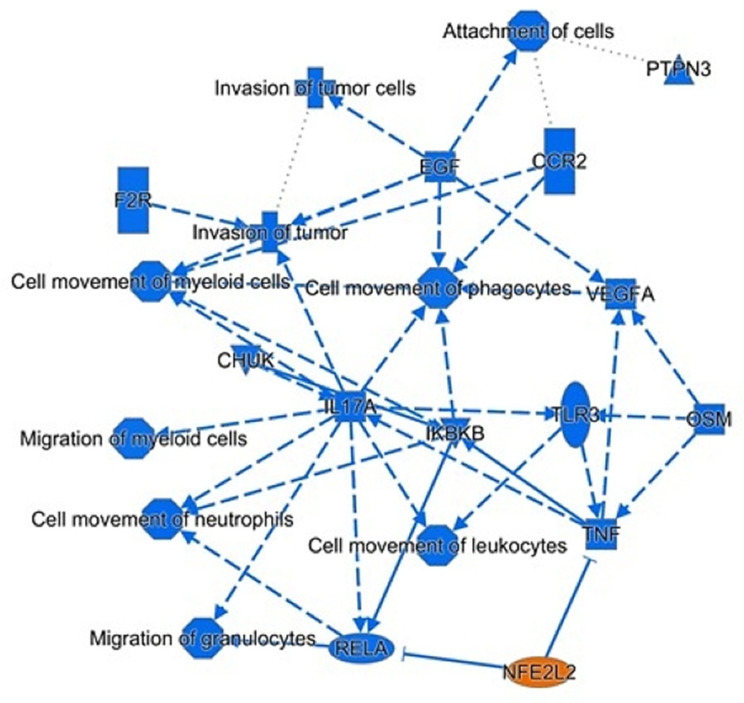
Graphical summary of IPA core analysis of differentially expressed proteins between Caki-1 and Caki-2 cells. The overview highlights the major biological themes in these analyses and their relationships connecting some of the most significant components predicted in the analysis. In this analysis, NRF2 (NFE2L2) is predicted to be significantly activated in Caki-2 cells (brown color). The major targets that are inhibited include TNF, RELA, and IKBKB. The result is the inhibition of multiple pathways that are associated with carcinogenesis.

Differentially expressed proteins between Caki-1 and Caki-2 cells were used in IPA analysis to determine top canonical pathways, molecular and cellular functions, and the top networks that are predicted to be associated with these proteins are summarized in Table 2. The summary of the analysis is depicted in Figure 3. Caki-2 cells were derived from a primary kidney tumor and these results predict the inhibition of molecules and pathways that drive metastasis and progression to aggressive disease phenotypes when compared to Caki-1 cells. These include inhibition of TNF, RELA, and VEGFA. We utilized IPA Upstream Regulator analysis to identify upstream transcriptional regulator cascades, explain the changes that are observed between the two cell lines, and uncover differences in biological activities occurring between the cells. In these analyses, NRF2, also known as the nuclear factor erythroid-derived 2-like 2 (NFE2L2), was predicted to be activated in Caki-2 cells but not in Caki-1 cells. This transcription factor was predicted to be activated and was identified with the highest activation *Z*-score of 3.65 and a significant *p*-value of overlap of 1.37 × 10^−20^.

**Figure 4:**
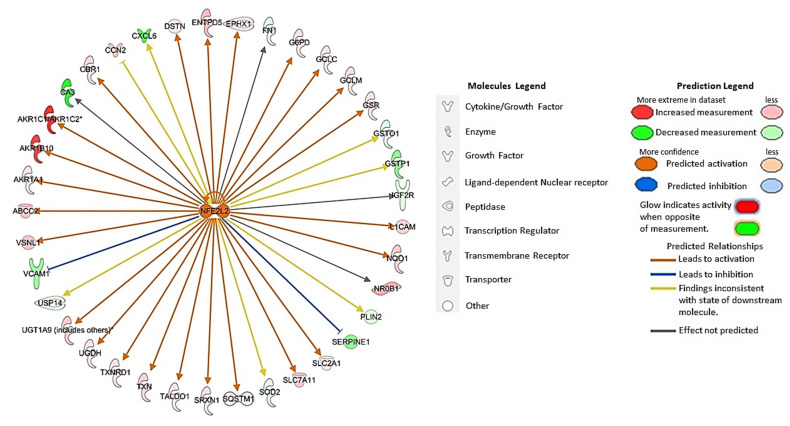
IPA upstream analysis network predicting the activation of NRF2 (NFE2L2). Proteins that are differentially expressed between Caki-1 and Caki-2 cells were subjected to IPA Upstream Regulator Analysis to identify upstream transcriptional regulators that can explain the observed protein expression changes in the two cells and predict the biological processes, pathways, and the upstream targets that they regulate. NRF2 is predicted to be activated and with the highest activation *Z*-score. The network predicts the activation and inhibition of specific targets with upregulation (e.g., AKR1B10, AKR1C1/AKR1C2 *), or downregulation (e.g., VCAM) of protein expression in Caki-2 cells, as observed in the quantitative proteomics analysis and subsequent validation by other biochemical approaches.

**Figure 5:**
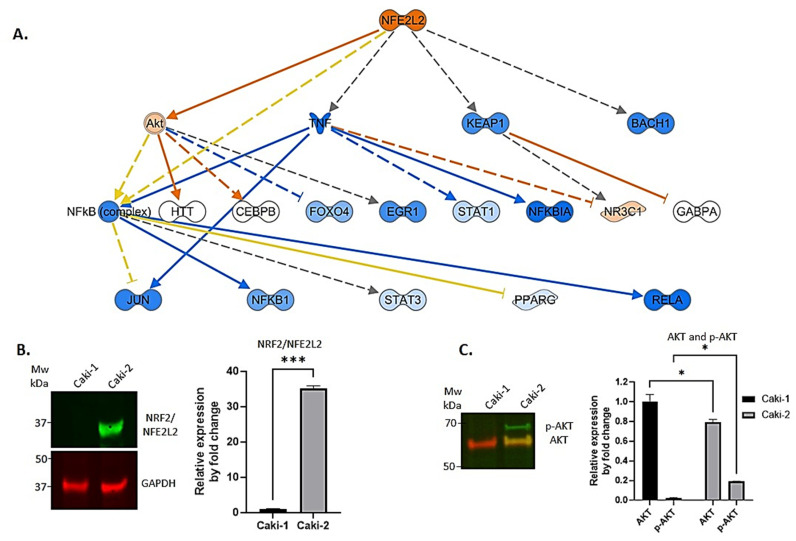
(**A**) Computationally generated IPA mechanistic pathway analysis network with plausible directional networks that are associated with the predicted activation of NRF2 (NFE2L2). In addition to NRF2 activation, among the targets that are predicted to be activated is AKT. As predicted, the immunoblot analysis demonstrates the activation and phosphorylation of AKT in Caki-2 cells but not in Caki-1 cells. (**B**)**.** Western blot analysis confirms the significant expression and upregulation of NRF2 in Caki-2 cells with a very low abundance of the protein detected in Caki-1 cells. (**C**). Activation of AKT is demonstrated by the significant increase in AKT phosphorylation in Caki-2 cells compared to Caki-1 cells. The pathway and network legends are identical to Figure 4. For the bar graphs, significant differential expression profiles are depicted using asterisks corresponding to the following thresholds * (*p* < 0.05) and *** (*p* < 0.001).

The upstream analysis network predicting the activation of NRF2 is shown in Figure 4 and the associated mechanistic pathway is depicted in Figure 5A. The predicted activation of NRF2 in Caki-2 cells was validated by Western blot (Figure 5B). These networks uncover several molecular nodes and molecular targets that may be crucial hubs for modulating critical functions that are unique in Caki-2 but not in Caki-1 cells, including the activation of AKT (Figure 5C).

**Figure 6:**
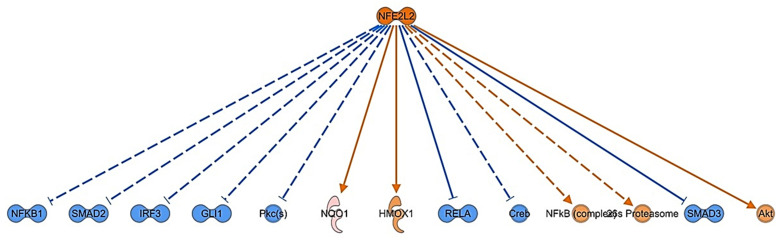
Computationally generated IPA causal network analysis of expanded upstream analysis, including regulators that may not be directly connected to differentially expressed proteins between Caki-1 and Caki-2 cells identifies NRF2 as the top hit. In addition to NRF2 activation, amongst the targets that are predicted to be activated (brown) are Akt, HMOX1, NFkB complex, and 26s proteasome activity. RELA and SMAD3 among others are predicted to be inhibited (blue). The pathway and network legends are identical to Figure 4.

**Figure 7:**
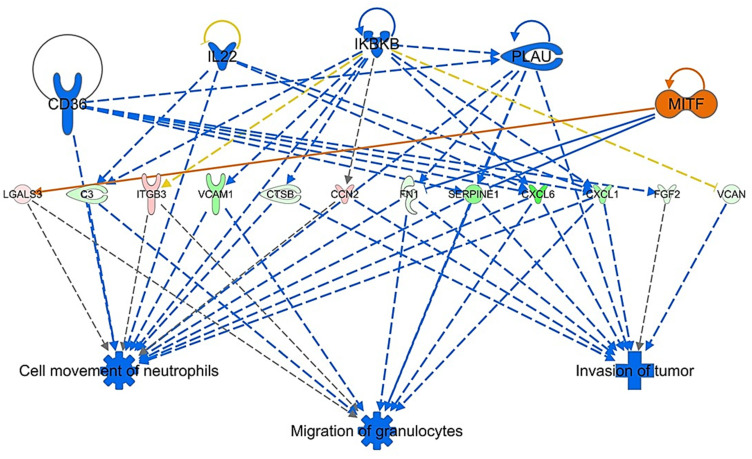
Top IPA Regulator Effect analysis network obtained after the integration of predicted upstream regulator results together with downstream effects to generate a causal hypothesis. There is predicted inhibition of four regulators including CD36, IL22, IKBK, and PLAU, whereas MITF is predicted to be activated in Caki-2 cells but not Caki-1 cells. The overall effect is predicted to inhibit the invasion of the tumor, cell moment of neutrophils, and migration of granulocytes.

IPA causal network analysis is used to uncover relationships that are associated with the differentially expressed proteins. This is achieved by expanding the upstream analysis to include regulators that are not directly connected to the identified targets. The top causal network identified includes activated NRF2 which is shown in Figure 6. The IPA Regulator Effect analysis algorithm is used to generate hypotheses to explain the role of the activation or inactivation of regulators and their consequences in mediating an increase or decrease of function and disease-related outcomes based on the evidence provided by a dataset (IPA, Qiagen). Regulator effect analysis of differentially expressed proteins between Caki-1 and Caki-2 cells predicted that the MITF microphthalmia-associated transcription factor (MITF) is activated in Caki-2 cells but not Caki-1 cells (Figure 7). The prediction of MITF activation is significant given that recently published data demonstrated that MITF expression was associated with aggressive tumor behavior and increased the migratory and invasive capabilities of ccRCC cells [46]. These results are novel with respect to the phenotype of Caki-2 cells and provide crucial insights into the histological classification of the cell line. The second highest activated transcription factor was MYC with a 2.784 activation *Z*-score and a *p*-value of overlap of 2.73 × 10^−13^, and the third was MAFA transcription factor MAFA) with a 2.449 activation Z-score and a *p*-value of overlap of 1.15 × 10^−4^.

The most significantly inhibited transcription regulator is predicted to be VHL with a Z-score of −2.700 and a *p*-value of overlap of 2.93 × 10^−10^. This is associated with the significant upregulation of CA12 in Caki-2 that is validated in these analyses. The next transcription regulator significantly inhibited is SP3 with a Z-score of −2.607 and a *p*-value of overlap of 2.87 × 10^−7^, followed by SMAD3 with a *p*-value of overlap of 1.22 × 10^−6^.

Casual network analysis identifies NRF2 as the top activated network with an activated *Z*-score of 4.007 and with a *p*-value of overlap of 5.92 × 10^−22^. In this network, molecules that are predicted to be activated include HMOX1, NQO1, and AKT. SALL4 is the transcription factor with the second-highest activation *Z*-score of 3.086 and a *p*-value of overlap of 3.53 × 10^−18^. The next transcription factor with the highest activated Z-score of 2.216 and a *p*-value of overlap of 1.42 × 10^−25^ is BTG2 (BTG family member two, also known as NGF-inducible antiproliferative protein PC3) which upon activation also leads to the activation of NRF2 and a subsequent inhibition of RELA and SMAD3. In the same analysis, BACH1 is predicted to be inhibited with an activation score of −3.862 and with a *p*-value of overlap of 4.36 × 10^−22^. The next transcription regulator predicted to be inhibited is NCOR1 with an activation *Z*-score of −2.964 and *p*-value of overlap of 3.53 × 10^−18^ and FOS with an activation *Z*-score of −2.926 and with a *p*-value of overlap of 2.88 × 10^−24^.

Additional bioinformatic analyses with the panel of 250 differentially expressed proteins were done using searches against the KEGG database [38], Wiki pathway database, and Reactome pathway database (https://reactome.org/) (accessed on 10 January 2023). The results for the top 10 enriched pathways using these three databases are provided in (Supplementary Table S5). These results are complementary, and some are concordant with the IPA data described in the preceding section.

## 4. Discussion

We have utilized a comprehensive high-performance tandem mass tag labeling-based mass-spectrometry approach to identify proteins that are differentially regulated in Caki-2 vs. Caki-1 cells [47]. Differentially regulated proteins were subsequently used to identify molecular pathways that are regulated and differentially activated in the two cell lines. We performed functional and pathway analyses to identify phenotypic differences in the two cell lines and the consequences of the differences in the pathogenesis of RCC. The most significantly upregulated proteins in Caki-2 vs. Caki-1 cells include members of the aldo-keto reductase enzymes superfamily, AKR1C2, AKR1C1, and AKR1B10. Functionally, human aldo-keto reductases play important roles in converting aldehydes and ketones to their corresponding alcohols using NADH and/or NADPH as cofactors [48,49,50,51,52]. Specifically, aldo-keto reductase family, members C1-C4 and D1 play central roles in the metabolism of steroid hormones, including androsterone and progesterone [48,51,53]. Previous studies focusing on their potential role in cancer biology [53,54,55,56], demonstrated a close relationship between AKR1C1 and sex hormone-related ovarian and bladder cancers [53,54,55,56,57]. A recent study focusing on identifying prognostic ferroptosis-related genes that are differentially regulated in KIRC cases in the TCGA database identified 12 genes that included AKR1C1 as a predictor for overall survival and is inferred to be related to immunity in ccRCC tumors [58,59]. On the other hand, AKR1B10, which belongs to the aldo-keto reductase (AKR) 1B subfamily, is involved in the reduction of aldehydes, some ketones and quinones, in addition to interacting with acetyl-CoA carboxylase and heat shock protein 90α [59]. In normal human tissues, AKR1B10 expression is predominantly observed in the alimentary canal [60]. AKR1B10 expression has also been observed in nonmalignant disease conditions including atopic dermatitis [61], diabetes [62], and leprosy [63]. The expression profile is different in multiple cancers and may be context dependent. In colon cancer, it has been characterized as a tumor suppressor gene [64], whereas it has been characterized as an oncogene in others, including pancreatic cancer [65], gastric cancer [66], hepatocellular carcinoma [67], and possibly nasopharyngeal cancer [68]. The significant overexpression of AKR1B10 in Caki-2 versus Caki-1, and its potential role in RCC, are unknown and warrant further investigations.

Our IPA current upstream analysis predicts the activation of the NRF2 (NFE2L2) in Caki-2 cells but not in Caki-1 cells. NRF2 was initially identified and characterized as a critical antioxidant response regulator and drug or xenobiotic detoxification, however, recent data demonstrate that it regulates multiple genes, including those that modulate proteasomal and autophagic function, iron, lipid and carbohydrate metabolism, amino acid metabolism, survival, proliferation, mitochondrial physiology, and DNA repair pathways [69]. The discovery of diverse pathways that involve NRF2 continues to happen, providing insights into its role in mediating disease progression and pathogenesis. These NRF2-mediated pathways can have protective and pathogenic consequences depending on the timing and duration of activation [70,71,72]. In cancer, abnormal NRF2 expression is involved in almost all the hallmarks associated with cancer, including progression, metastasis, and drug resistance [70,73,74,75,76]. Data from the Human Protein Atlas [77], demonstrates differential expression of NRF2 in KIRC, KIRP, and KICH (https://www.proteinatlas.org/ENSG00000116044-NFE2L2/pathology/renal + cancer). The survival probabilities are shown in Supplementary Figure S2A–C. It is notable that higher expression levels of NRF2 correlate with significantly higher survival rates in KIRC (*p* = 9.8 × 10^−7^) but not in KIRP, where low expression is associated with higher survival rates (*p* = 0.029). By contrast, the survival rates of patients with high and low NRF2 expression in KICH are not significantly different. In the IPA analysis, the activation of NRF2 is predicted to directly inhibit TNF and RELA, which subsequently leads to the downregulation of IKBKB and RELA amongst other molecules. The net result of these processes includes inhibition of invasion of tumor cells, attachment of cells, cell movement, and migration of immune cells. These observations would imply a tumor suppressor role for Caki-2 which was isolated from a primary tumor (nonaggressive) compared to Caki-1 which was isolated from a distant-skin metastatic site and with a defined aggressive ccRCC phenotype.

In addition, we have used the TCGA database to uncover the clinical relevance of some of the significantly differentially regulated proteins between CAKI-1 and CAKI-2 cells to determine if their expression profiles correlate with patient disease state and five-year survival rates. In the case of CA12, we have validated the MS data showing significant upregulation of the protein in Caki-2- cells versus Caki-1 cells using orthogonal methods including immunoblotting, qPCR, immunofluorescence confocal microscopy, and confirmed the differential sensitivity of Caki-2 cells to S4 inhibitor under normoxic and hypoxic conditions as described in the results section. Five-year TCGA survival analysis data shows significant differences between low and high *CA12* expression patients (*p* = 0.0070) (Supplementary Figure S3A–C) https://www.proteinatlas.org/ENSG00000074410-CA12/pathology/renal+cancer (accessed on 13 March 2023). The five-year survival rate for high expressers is 55% versus 67% for low expressers in ccRCC patients, though there were no statistical differences in five-year survival rates for pRCC (*p* = 0.072) and chRCC (*p* = 0.25) patients. Further analysis shows significant differences between the expression profiles of *CA12* between KIRC, KIRP, and KICH tumor types of cells with an ANOVA F-test with a value of *p* = 1.021 × 10^−79^ (Supplementary Figure S3D). These observations would imply a potential prognostic and therapeutic role for CA12 in a subset of ccRCC cases, which is currently the subject of our further investigation. A similar analysis focuses on *IGF2BP1* which is significantly upregulated with a 30-fold increase in Caki-1 cells versus Caki-2 cells. The five-year TCGA survival analysis data shows significant differences between low and high *IGF2BP1* expression patients (*p* = 0.0015) (Supplementary Figure S4A–C) https://www.proteinatlas.org/ENSG00000159217-IGF2BP1/pathology/renal+cancer (accessed on 15 March 2023). The five-year survival rate for high expressers is 57% versus 71% for low expressers in ccRCC patients. Significant differences also exist in the five-year survival rates for pRCC (*p* = 0.00011) and chRCC (*p* = 0.00092) patients. Further analysis shows significant differences between the expression profiles of *IGF2BP1* between KIRC, KIRP, and KICH tumor types cells with an ANOVA F-test with a value of *p* = 6.615 × 10^−18^ (Supplementary Figure S4D). These observations would imply a potential oncogenic role in Caki-1 cells, though not in Caki-2 cells. IGF2BP1 has been reported as a potential prognostic marker for RCC [78,79].

## 5. Conclusions

Our results indicate that multiple proteins and molecular pathways are differentially regulated and activated in Caki-2 versus Caki-1 cells. The transcription factor NRF2, encoded by the *NFE2L2* gene is the most significantly activated transcription factor between the two cell lines based on activation *Z*-scores. The gene is highly activated in Caki-2 cells compared to Caki-1 cells. NRF2 mediates complex regulatory pathways, playing a pleiotropic role in regulating metabolism, mitochondrial physiology, autophagy, proteostasis, inflammation, and immune responses. Although the regulation of NRF2 expression in RCC is incompletely understood and warrants further investigation, our data suggest that Caki-2 RCC cells that express high NRF2 levels are predicted to show inhibition of immune cells, inhibition of migration and invasion of tumor and tumor cells, and TNF and RELA inhibition. The activation of NRF2 in Caki-2 vs. Caki-1 cells requires further investigation to uncover its role in the initiation and progression of disease in different ccRCC cell line subtypes. These findings provide new insights that may provide the basis for developing targeted therapeutics for molecular subtypes of RCC that are mediated by the functions of NRF2.

## Figures and Tables

**Table 1:** Comparative quantitative proteomic analysis between Caki-1 and Caki-2 cells. The top 15 proteins which were upregulated in Caki-2 (down-regulated in Caki-1) have positive expression values. The top 15 proteins downregulated in Caki-2 (upregulated in Caki-1) have negative expression values.

Gene Symbol	Protein Accession	Abundance Ratio (Caki-2/Caki-1).	Gene Symbol	Protein Accession	Abundance Ratio (Caki-2/Caki-1)
*AKR1C2*	P52895	37.660	*TCEAL3*	Q969E4	−50.000
*CA12*	O43570	28.951	*CA3*	P07451	−37.037
*TPPP3*	Q9BW30	27.533	*TPM2*	P07951	−37.037
*AKR1B10*	O60218	26.012	*ADAMTSL1*	Q8N6G6	−33.333
*PDZK1*	Q5T2W1	25.480	*YLPM1*	P49750	−33.333
*FABP6*	P51161	24.132	*IGF2BP1*	Q9NZI8	−32.258
*CYP2S1*	Q96SQ9	23.136	*VAMP5*	O95183	−30.303
*SNCG*	O76070	22.877	*CXCL6*	P80162	−30.303
*SFN*	P31947	21.316	*PRDX2*	P32119	−27.778
*SPINT2*	O43291	21.130	*FBN2*	P35556	−25.000
*SERPINB5*	P36952	19.572	*BASP1*	P80723	−25.000
*NEFL*	P07196	17.122	*BCAT1*	P54687	−22.222
*GLDC*	P23378	16.902	*TRIM22*	Q8IYM9	−21.277
*NAPRT*	Q6XQN6	16.742	*SERPINE1*	P05121	−19.608
*TYMP*	P19971	16.403	*GSTP1*	P09211	−19.231

**Table 2:** Summary of IPA Analysis of differentially expressed proteins between Caki-1 and Caki-2 cells. The top five canonical pathways, the top five molecular and cellular functions, and the top five networks and their functions are provided.

Top Canonical Pathways		
Name	*p*-Value	Overlap
NRF2-mediated Oxidative Stress Response	5.76 × 10^−11^	7.6% 18/237
LPS/IL-1-mediated Inhibition of RXR Function	1.40 × 10^−9^	6.7% 17/254
Xenobiotic Metabolism AHR Signaling Pathway	2.29 × 10^−8^	11.5% 10/87
Xenobiotic Metabolism CAR Signaling Pathway	1.04 × 10^−7^	6.8% 13/191
Xenobiotic Metabolism PXR Signaling Pathway	1.10 × 10^−7^	6.8% 13/192
Molecular and Cellular Functions		
Name	*p*-value range	# Molecules
Cellular Movement	1.46 × 10^−6^–9.26 × 10^−25^	117
Cell Death and Survival	6.02 × 10^−7^–3.90 × 10^−21^	128
Cell Morphology	8.27 × 10^−7^–1.04 × 10^−17^	71
Cell-To-Cell Signaling and Interaction	1.53 × 10^−6^–1.46 × 10^−15^	84
Cellular Development	1.59 × 10^−6^–4.45 × 10^−14^	123
Top Networks		
Associated Network Functions		Score
Cellular Assembly and Organization, Cellular Comprise, Cellular Function, and Maintenance		60
Cell-To-Cell Signaling and Interaction, Cancer, Endocrine System Disorders		46
Cellular Assembly and Organization, Cell Morphology, Cellular Movement		41
Drug Metabolism, Liver Hyperbilirubinemia, Metabolomics Disease		32
Vitamin and Mineral Metabolism, Carbohydrate Metabolism, Drug Metabolism		27

## Data Availability

The mass spectrometry proteomics data have been deposited to the ProteomeXchange Consortium via the PRIDE partner repository with the data set identifier PXD040083.

## Supplementary Materials

The following information can be downloaded at: https://www.mdpi.com/article/10.3390/biomedicines11041010/s1, Figure S1: Differential expression of HIF1A in Caki-1 and Caki-2 cells. Figure S2: Differential expression of *NRF2* in ccRCC, pRCC and chRCC. Figure S3: Differential expression of *CA12* in ccRCC, pRCC and chRCC. Figure S4: Differential expression of *IGF2BP1* in ccRCC, pRCC and chRCC. Table S1: Origin and characteristics of Caki-1 and Caki-2 cells. Table S2: MS method summary. Table S3: Antibodies used in the study. Table S4: List of differentially expressed proteins between Caki-2 and Caki-1 cells. Table S5: Enrichment pathways.
