# Supplementary material for: Differential Activation of NRF2 Signaling Pathway in Renal-Cell Carcinoma Caki Cell Lines
Source: Biomedicines. 2023 Mar 24;11(4):1010. doi: 10.3390/biomedicines11041010 (PMC10135738; doi:10.3390/biomedicines11041010)

biomedicines

Supplementary Table S1.

Cell Line	Origin	Characteristics
Caki-1	Metastatic clear cell renal cell carcinoma cells isolated from skin lesion of 49 year old Caucasian male	Isolated from skin metastatic lesion. Tumorigenic, forms clear cell carcinoma in nude mice consistent with renal primary.
Caki-2	Clear cell carcinoma cells isolated from a 69 year old Caucasian American male primary tumor	Isolated from a primary tumor. Tumorigenic, forms clear cell carcinoma in nude mice.

Supplementary Table S2. MS Method Summary

	OT-MS3
**MS1**	
mass analyzer	Orbitrap
resolution	120K
scan range	400–1400
AGC target	4.0×10^5
max. injection time (ms)	50
RF lens (%)	30
**MS2**	
charge states	2–6
mass analyzer	ion trap
activation type	CID
dynamic exclusion	cycle time
isolation window	0.4
collision energy	35
scan rate/resolution	turbo
AGC target	1.0×10^4
max. injection time (ms)	50
**MS3**	
number of SPS ions	10
isolation window	1.2
activation type	HCD
collision energy	65
mass analyzer	Orbitrap
scan rate/resolution	7.5K
scan range (Th)	"normal"
AGC target	1.5×10^5
max. injection time (ms)	150

Supplementary Table S3. Antibodies

Target	Species	Vendor	Catalogue #	Dilution
AKR1C1	mouse monoclonal	Santa Cruz Biotechnology	sc-166297	1:500
AKT	rabbit polyclonal	Cell Signaling Technologies	Cat. # 9272	1:500
p- AKT	mouse monoclonal	Cell Signaling Technologies	Cat. # 4051S	1:500
CAIX (CA9)	mouse monoclonal	Santa Cruz Biotechnology	sc-365900	1:500

CAXII (CA12)	mouse monoclonal	Santa Cruz Biotechnology	sc-374314	1:500
GAPDH	rabbit polyclonal	Santa Cruz Biotechnology	sc-25778	1:1000
HiF1α	mouse monoclonal	Santa Cruz Biotechnology	sc-13515	1:500
NEFL	mouse monoclonal	Santa Cruz Biotechnology	sc-390732	1:500
Nrf2	mouse monoclonal	Santa Cruz Biotechnology	sc-365949	1:500
VHL	mouse monoclonal	Santa Cruz Biotechnology	sc-15657	1:500
anti-Rabbit IgG	donkey	LI-COR	926-68073	1:20,000
anti-Mouse IgG	donkey	LI-COR	926-32212	1:20,000

Supplementary Table S4. List of differentially expressed proteins between Caki-2 and Caki-1 cells.

Supplementary Table S5.

Enrichment KEGG Pathway database

	Accession	Description	Size	Overlap	Expected	Fold Enrichment	p-value	-log 2 (p-Value)
1	hsa00053	Ascorbate and aldarate metabolism	16	8	1.3	5.95	1.67e-05	15.87
2	hsa00071	Fatty acid degradation	31	11	2.6	4.22	2.41e-05	15.34
3	hsa05204	Chemical carcinogenesis	27	10	2.3	4.41	3.7e-05	14.72
4	hsa00410	Beta-Alanine metabolism	17	6	1.4	4.2	0.0019	9.04
5	hsa00531	Glycosaminoglycan degradation	8	4	0.7	5.95	0.00263	8.57
6	hsa00790	Folate biosynthesis	13	5	1.1	4.57	0.00301	8.38
7	hsa00340	Histidine metabolism	10	4	0.8	4.76	0.00689	7.18
8	hsa04061	Viral protein interaction/cytokines	11	4	0.9	4.33	0.0101	6.63
9	hsa00910	Nitrogen metabolism	7	3	0.6	5.1	0.016	5.97
10	hsa00604	Glycosphingolipid biosynthesis	4	2	0.3	5.95	0.0378	4.73

Enrichment Wikipathways database

	Accession	Description	Size	Overlap	Expected	Fold Enrichment	p-value	-log 2 (p-Value)
1	WP98	Prostaglandin synthesis and regulation	16	10	1.3	7.43	8.27e-08	23.53
2	WP696	Benzo(a)pyrene metabolism	6	5	0.5	9.91	2.31e-05	15.49
3	WP692	Sulfation biotransformation reaction	8	5	0.7	7.43	0.000187	12.39
4	WP3934	Leptin and adiponectin	5	4	0.4	9.52	0.000231	12.08
5	WP1272	Selenium micronutrient network	3	3	0.3	11.9	0.000592	10.72
6	WP1310	Selenium micronutrient network	3	3	0.3	11.9	0.000592	10.72
7	WP3891	Benzene metabolism	2	2	0.2	11.9	0.00706	7.15
8	WP206	Fatty acid omega-oxidation	2	2	0.2	11.9	0.00706	7.15
9	WP1080	Irinotecan pathway	3	2	0.3	7.93	0.02	5.64
10	WP4228	Vitamin B6-dependent disorders	3	2	0.3	7.93	0.02	5.64

Enrichment Reactome pathways database

	Accession	Description	Size	Overlap	Expected	Fold Enrichment	p-value	-log 2 (p-Value)
1	R-HAS-9759194	Nuclear events mediated by NFE2L2	18	13	1.5	8.95	6.29e-11	33.89

2	R-HAS-380108	Chemokine receptors binding chemokines	6	4	0.5	7.83	0.000647	10.59
3	R-HAS-444473	Formyl peptide receptors binding formyl peptides	4	3	0.3	8.92	0.00222	8.82
4	R-HAS-4717374	Defective DPM1 causes DPM1-CDG	2	2	0.2	11.9	0.00706	7.15
5	R-HAS-163574	Insulin effects increased synthesis of Xylulose-5-phosphate	2	2	0.2	11.9	0.00706	7.15
6	R-HAS-162699	Synthesis of dolichy-phosphate mannose	2	2	0.2	11.9	0.00706	7.15
7	R-HAS-4719360	Defective DPM3 causes DPM3-CDG	2	2	0.2	11.9	0.00706	7.15
8	R-HAS-4719377	Defective DPM3 causes DPM3-CDG	2	2	0.2	11.9	0.00706	7.15
9	R-HAS-392154	Nitric oxide stimulates guanylate cyclase	2	2	0.2	11.9	0.00706	7.15
10	R-HAS-5578999	Defective GCLC causes HAGGSD	2	2	0.2	11.9	0.00706	7.15

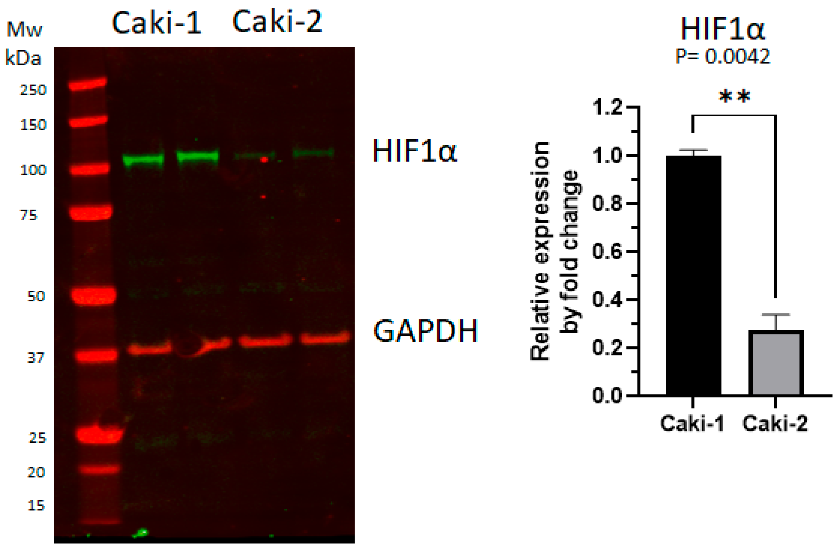

Supplementary Figure S1. Differential expression of HIF1A in Caki-1 and Caki-2 cells. Western blot detection of HIF1*α* in Caki-1 and Caki-2 cells. GAPDH was used as a loading control and expression fold change of the proteins are based on normalization by individual GAPDH values. HIF1*α* is significantly upregulated in Caki-1 compared to Caki-2 cells.

Supplementary Figure S2. Data from the Human Protein Atlas demonstrates differential expression of NRF2 in Kidney Renal Clear Cell Carcinoma (KIRC), Kidney Papillary Cell Carcinoma (KIRP), and Kidney Chromophobe Carcinoma (KICH). The survival probabilities scores and 5 year survival rates are shown in panel **(A).** KIRC, **(B)** KIRP and, **(C)** KICH. https://www.proteinatlas.org/ENSG00000116044-NFE2L2/pathology/renal+cancer

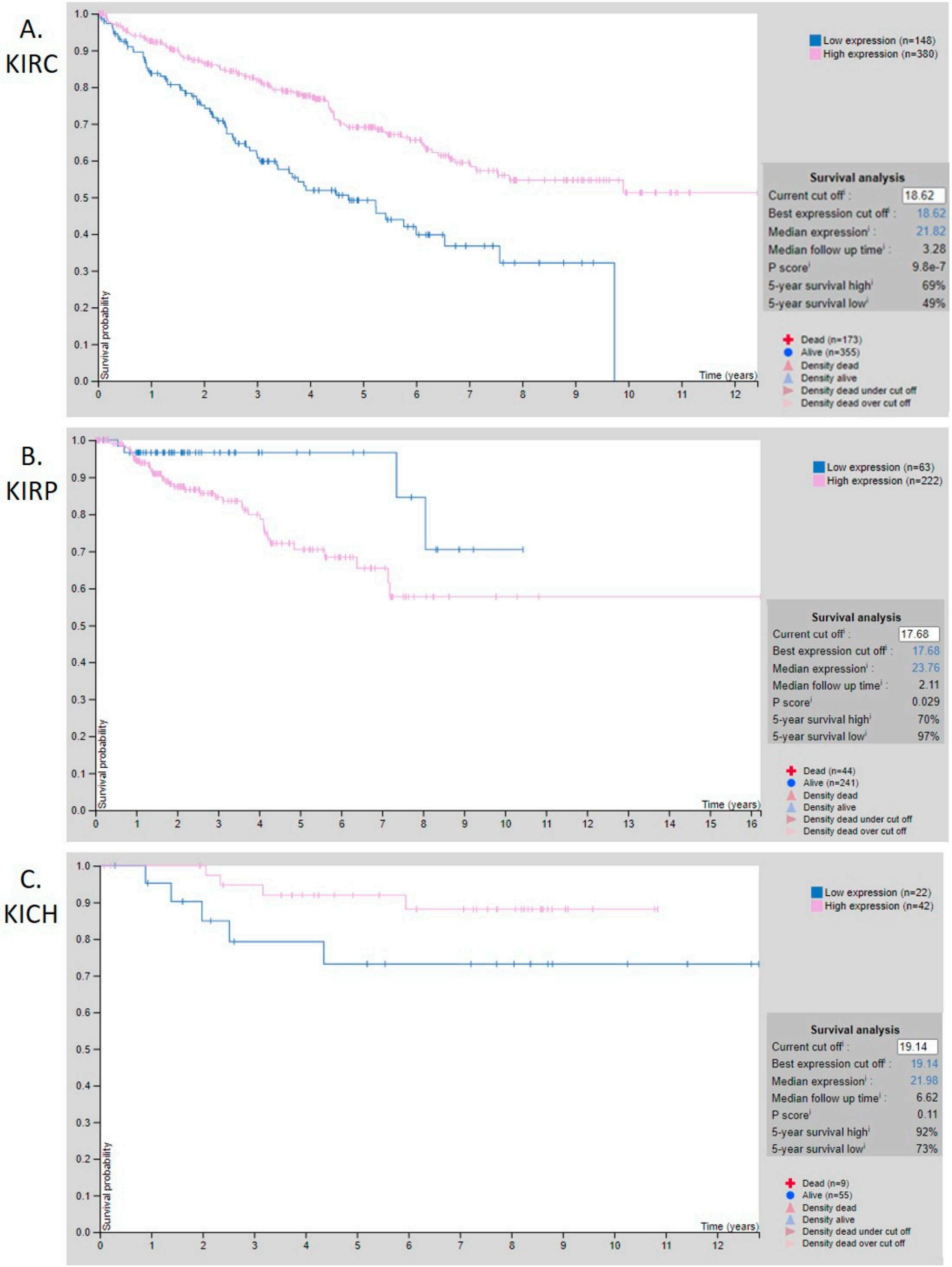

Supplementary Figure S3. **The Cancer Genome Atlas (TCGA) demonstrates differential expression of *CA12* in Kidney Renal Clear Cell Carcinoma (KIRC), Kidney Papillary Cell Carcinoma (KIRP), and Kidney Chromophobe Carcinoma (KICH).** The survival probabilities scores and 5 year survival rates are shown in panel **(A).** KIRC, **(B)** KIRP and, **(C).** KICH. Additional *CA12* FPKM information for the three tumor types is provided in panel **(D)**.

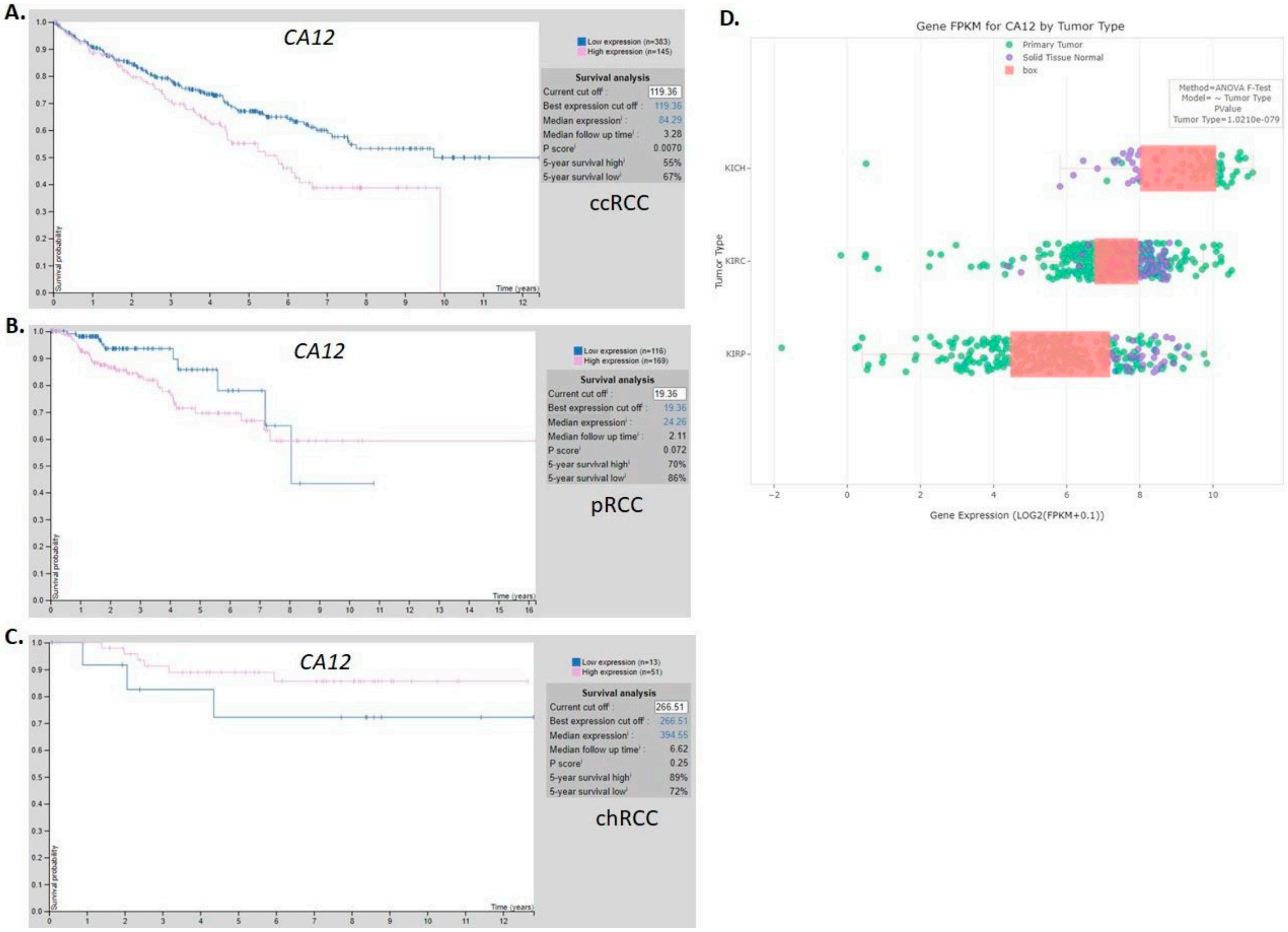

Supplement: Supplementary file 1 [file biomedicines-11-01010-s001.zip › biomedicines-2248930-supplementary.pdf]
